# Cranial and spinal computed tomography (CT) angiography with photon-counting detector CT: comparison with angiographic and operative findings

**DOI:** 10.1007/s11604-024-01661-w

**Published:** 2024-09-16

**Authors:** Fumiyo Higaki, Masafumi Hiramatsu, Takao Yasuhara, Susumu Sasada, Yoshihiro Otani, Jun Haruma, Tomohiro Inoue, Yusuke Morimitsu, Noriaki Akagi, Yusuke Matsui, Toshihiro Iguchi, Takao Hiraki

**Affiliations:** 1 Department of Radiology, Okayama University Hospital, 2-5-1 Shikata-cho, Kita-ku, Okayama, Japan; 2 Department of Neurological Surgery, Faculty of Medicine, Dentistry, and Pharmaceutical Sciences, Okayama University, 2-5-1 Shikata-cho, Kita-ku, Okayama, Japan; 3 Division of Radiological Technology, Okayama University Hospital, 2-5-1 Shikata-cho, Kita-ku, Okayama, Japan; 4 Department of Radiology, Faculty of Medicine, Dentistry, and Pharmaceutical Sciences, Okayama University, 2-5-1 Shikata-cho, Kita-ku, Okayama, Japan; 5 Department of Radiological Technology, Faculty of Health Sciences, Okayama University, 2-5-1 Shikata-cho, Kita-ku, Okayama, Japan

**Keywords:** Photon-counting detector CT, Energy-integrating detector CT, CT angiography, Neuroimaging, Spine

## Abstract

The clinical imaging features of photon-counting detector (PCD) computed tomography (CT) are mainly known as dose reduction, improvement of spatial resolution, and reduction of artifacts compared to energy-integrating detector CT (EID-CT). The utility of cranial and spinal PCD-CT and PCD-CT angiography (CTA) has been previously reported. CTA is a widely used technique for noninvasive evaluation. Cranial CTA is important in brain tumors, especially glioblastoma; it evaluates whether the tumor is highly vascularized prior to an operation and helps in the diagnosis and assessment of bleeding risk. Spinal CTA has an important role in the estimation of feeders and drainers prior to selective angiography in the cases of spinal epidural arteriovenous fistulas and spinal tumors, especially in hemangioblastoma. So far, EID-CTA is commonly performed in an adjunctive role prior to selective angiography; PCD-CTA with high spatial resolution can be an alternative to selective angiography. In the cases of cerebral aneurysms, flow diverters are important tools for the treatment of intracranial aneurysms, and postoperative evaluation with cone beam CT with angiography using diluted contrast media is performed to evaluate stent adhesion and in-stent thrombosis. If CTA can replace selective angiography, it will be less invasive for the patient. In this review, we present representative cases with PCD-CT. We also show how well the cranial and spinal PCD-CTA approaches the accuracy of angiographic and intraoperative findings.

## Introduction

A long history of photon-counting detector (PCD) computed tomography (CT) development has been reported [1–5]. Long after the first prototype PCD-CT by GE Healthcare in 2008, a first-generation clinical PCD-CT with a cadmium telluride (CdTe) detector (NAEOTOM Alpha; Siemens Healthcare) has become commercially available since 2021. The clinical imaging features of PCD-CT are mainly dose reduction, improvement of spatial resolution, and reduction of artifacts compared to energy-integrating detector CT (EID-CT) [6–8]. Specifically, in cranial CT, PCD-CT image quality scores were significantly higher, with lower image noise and fewer artifacts compared to those of EID-CT [9]. The reported improvement rates for PCD-CT compared to those for EID-CT were 12.8–20.6% for image noise, 19.0–20.0% for signal-to-noise ratio, 15.7% for gray matter (GM)–white matter contrast (WM), and 33.3% for GM–WM contrast-to-noise ratio (CNR) [10]. In cranial CT angiography (CTA), precise lumen visualization and depiction of metallic stent meshes with PCD-CT systems with higher accuracy than EID-CT were reported [11]. PCD-CTA after flow diverter implantation for an internal carotid artery aneurysm shows slight fish-mouthing at the proximal end of the stent, and images are presented to assess incomplete attachment to the medial curve [11]. PCD-CT after venous stenting shows detailed visualization of stent structure and excellent demonstration of stent intimal hyperplasia [11]. Spinal PCD-CT provides a significantly lower radiation dose for lumbar spine evaluation compared to EID-CT, maintaining image quality of trabecular architecture, cortical bone, paraspinal muscles, and intervertebral disc [12].

In this review, we present previous PCD-CT reports and an overview of the disease for representative cases. We also show how well the cranial and spinal CTA with PCD-CT approaches the accuracy of angiographic and intraoperative findings.

## Features of PCD-CT and differences in comparison with EID-CT

The important difference between EID-CT and PCD-CT is that EID-CT involves a two-step detection process, while PCD-CT has a one-step direct X-ray conversion process. The two-step detection process of EID-CT is performed using a solid scintillator detector consisting of gadolinium oxysulfide (Gd_2_O_2_S) or cadmium tungstate (CdWO_4_) to convert X-ray energy into visible light, which is then converted into an electronic signal. X-rays that have various energy spectra are combined into a single measurement when converted to light. In contrast, in PCD-CT, when an X-ray photon enters the detector, it interacts with the detector to produce an electron–hole pair. The charges are separated by a strong electric field between the cathode at the top of the detector and the pixelated anode at the bottom, generating a pulse; ideally, one charge cloud generated by one photon enters one pixel electrode, generating one pulse. However, charge-sharing, in which a charge cloud created by one photon is collected at two anodes and creates two pulses, is a technical issue. There is also pulse pile-up, a phenomenon in which multiple photons injected at almost the same time are counted as one. PCD-CT uses a detector with a large atomic number and high X-ray absorption efficiency, such as CdTe. Furthermore, unlike EID, PCD does not require a detector separation wall, resulting in higher dose efficiency. PCD-CT can eliminate electronic noise if the signal to the detector is small. Moreover, in a PCD-CT scan, all photons are counted equally from low to high energy. Compared to EID, the features of PCD lead to better spatial resolution, higher contrast-to-noise ratio, electron noise rejection, and improved dose efficiency. These characteristics have been reported in previous reviews [6–8].

## Clinical neuroimaging

Some previous papers on cranial and spinal PCD-CT imaging (in vivo, human) are listed in Table 1. PCD-CT provides superior image quality with significantly lower noise and fewer artifacts compared to EID-CT [9–12].

**Table 1:** Previous reports of cranial and spinal PCD-CT imaging

Author	Year		Result
Pourmorteza et al. [10]	2017	Cranial	PCD-CT demonstrated greater gray–white matter contrast due to higher soft-tissue contrast and lower image noise compared to EID-CT
Symons et al. [9]	2018	Cranial	PCD-CT image quality scores were significantly higher with lower image noise and fewer artifacts compared to EID-CT images
Abel et al. [11]	2023	Cranial	PCD-CTA provides precise lumen visualization and depiction of metallic stent meshes with higher accuracy than EID-CTA
Marth AA et al. [12]	2024	Spine	PCD-CT provides significantly lower radiation dose for lumbar spine evaluation compared to EID-CT

*CT* computed tomography, *PCD-CT* photon-counting detector CT, *EID-CT* energy-integrating detector CT

CTA is a widely used technique for noninvasive evaluation. Cranial CTA is important in brain tumors, especially glioblastomas; it evaluates whether the tumor is highly vascularized prior to operation and helps with the diagnosis and assessment of bleeding risk during operation. Spinal CTA has an important role in the estimation of feeders and drainers prior to selective angiography in the cases of spinal epidural arteriovenous fistulas and spinal tumors, especially in hemangioblastomas. So far, EID-CTA is commonly performed in an adjunctive role prior to selective angiography; PCD-CTA with high spatial resolution can be an alternative to selective angiography. In the cases of cerebral aneurysms, flow diverters are important tools for the treatment of intracranial aneurysms [13, 14], and postoperative evaluation by angiography with cone beam CT with improved spatial resolution [15] is widely performed to evaluate stent adhesion and in-stent thrombosis. Although cranial and spinal CTA cannot replace cerebral angiography in the evaluation of hemodynamics, it can be alternatively used to evaluate vascular architectures. Furthermore, as cerebral angiography is associated with complications, such as cerebral infarction, its substitution with CTA is beneficial for patients.

In addition to evaluating tumor vascularity, CT has a role in assessing tumor calcification for diagnosis and preoperative evaluation.

In this review, we present representative cases with PCD-CT, and we show how well the cranial and spinal PCD-CTA approaches the accuracy of angiographic and intraoperative findings.

### Acquisition and imaging

Acquisition and imaging parameter settings of the PCD-CT system were as follows: for cranial CTA—tube voltage 140 kV, tube current Eff. mAs 161, collimation 120 × 0.2 mm, rotation time 0.25 s, pitch 0.6, kernel Hv64 Hv89, and image matrix 512. For spinal CTA, the settings were as follows: tube voltage 120 kV, tube current Eff. mAs 270, collimation 120 × 0.2 mm, rotation time 0.5 s, pitch 0.85, kernel Qr44 Qr60, and image matrix 512. Automatic tube current modulation was used for dose optimization in patients.

Test injection method was used for cranial CTA. The test injection was performed using 10 ml of contrast and 20 ml of saline. The contrast arrival time was measured by monitoring the internal carotid artery at the level of the infraorbital border. The acquisition start time was 2 s after the peak of the time enhancement curve. The conditions for contrast injection in cranial CTA were as follows: the fractional dose was set at 28 mgI/kg/s, and the contrast was injected for 12 s along with 30 ml of saline. The bolus tracking method was used for spinal CTA. The conditions for contrast injection in spinal CTA were as follows: the fractional dose was set at 28 mgI/kg/s, and the contrast was injected for 30 s along with 40 ml of saline. Monitoring was performed at the Th10 level, and the scan began 6 s after the CT value of the abdominal aorta reached 250 HU.

### Spinal epidural arteriovenous fistula (SEAVF)

Thoracolumbar and sacral SEAVFs are increasingly being recognized as a form of spinal vascular malformation. SEAVFs are subclassified as type A (with intradural drainage of shunt flow) and type B (without intradural drainage) [16, 17].

A systematic review [18] of thoracolumbar SEAVF indicated a mean age of 63.5 years among patients, of whom 69.6% were male. Sensory symptoms, such as pain and numbness, were the most common manifestation. The site of the fistula was the lumbosacral and thoracic spines in 79.2% and 20.8% of patients, respectively.

Endovascular and microsurgical treatments are associated with favorable clinical outcomes along with high occlusion rates. However, SEAVFs are often misdiagnosed [19] as spinal dural arteriovenous fistulas (SDAVFs), and an accurate understanding of the vascular architecture is important for determining the treatment strategy and obviating recurrence.

Kiyosue et al. [19] reported similarities and differences between SEAVFs and SDAVFs. Both are most common in patients in their 60 s and are associated with progressive myelopathy. SDAVFs are more common than SEAVFs at the thoracic level, and SEAVFs are more common than SDAVFs at the lumbar level. SDAVFs are more associated with a history of trauma and spinal surgery than SEAVFs. The main feeding artery in an SDAVF is often a radiculomeningeal artery, whereas SEAVFs frequently have a ventral and dorsal somatic branch vascular supply, in which an epidural shunted pouch is formed.

To diagnose SEAVF, selective angiography of the segmental artery is performed. In patients with normal renal function, CTA is performed prior to selective angiography to determine the branching artery that generates the feeder artery.

**Fig. 1:**
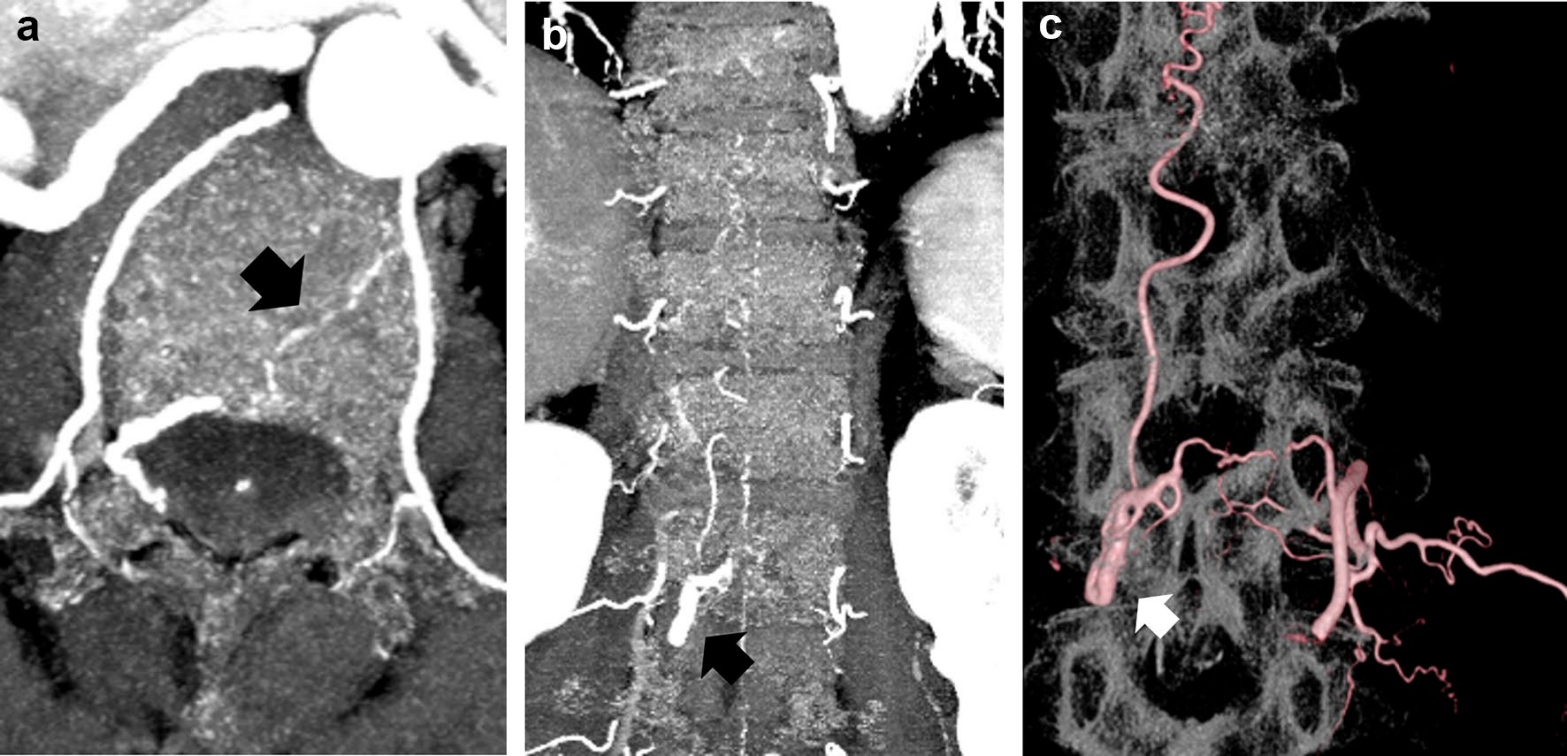
Spinal epidural arteriovenous fistula (AVF). A case of a female patient in her 60 s with complaints of numbness, coldness, and stiffness of both lower extremities for several months. On magnetic resonance imaging (MRI), the T1-weighted images (T1WI) and T2-weighted images (T2WI) showed extensive edema in the spinal cord and multiple flow voids on the surface of the spinal cord. The PCD-CTA (axial image: kernel Qr60) shows the ventral somatic branch (arrow), which branches from the L1 segmental artery, internally traverses the vertebral body, and joins into the epidural shunted pouch. The epidural shunted pouch continues with the radiculomedullary vein and refluxes into the posterior spinal veins. Comparison of the PCD-CTA images (**a**, **b**) with selective angiography coronal view (**c**) shows that the L1 segmental artery, the ventral somatic branch (**a**, arrow), the shunted pouch (**b**, arrow), and the ascending retrograde radiculomedullary vein were almost equally well delineated. *PCD-CTA* photon-counting detector-computed tomography angiography

We present a representative case of an SEAVF with a feeding artery that traverses into the bone, which was identified using PCD-CTA and selective catheter spinal angiography (Fig. 1).

### Cerebral aneurysm treated with flow diverter

Flow diverter stents are important tools for the treatment of intracranial aneurysms. Meta-analyses suggest that flow diverters are an effective treatment of intracranial aneurysms with high complete occlusion rates [20]. In general, however, there are cases where aneurysm occlusion cannot be obtained, and occlusion may be difficult in cases where stent apposition has not been obtained.

**Fig. 2:**
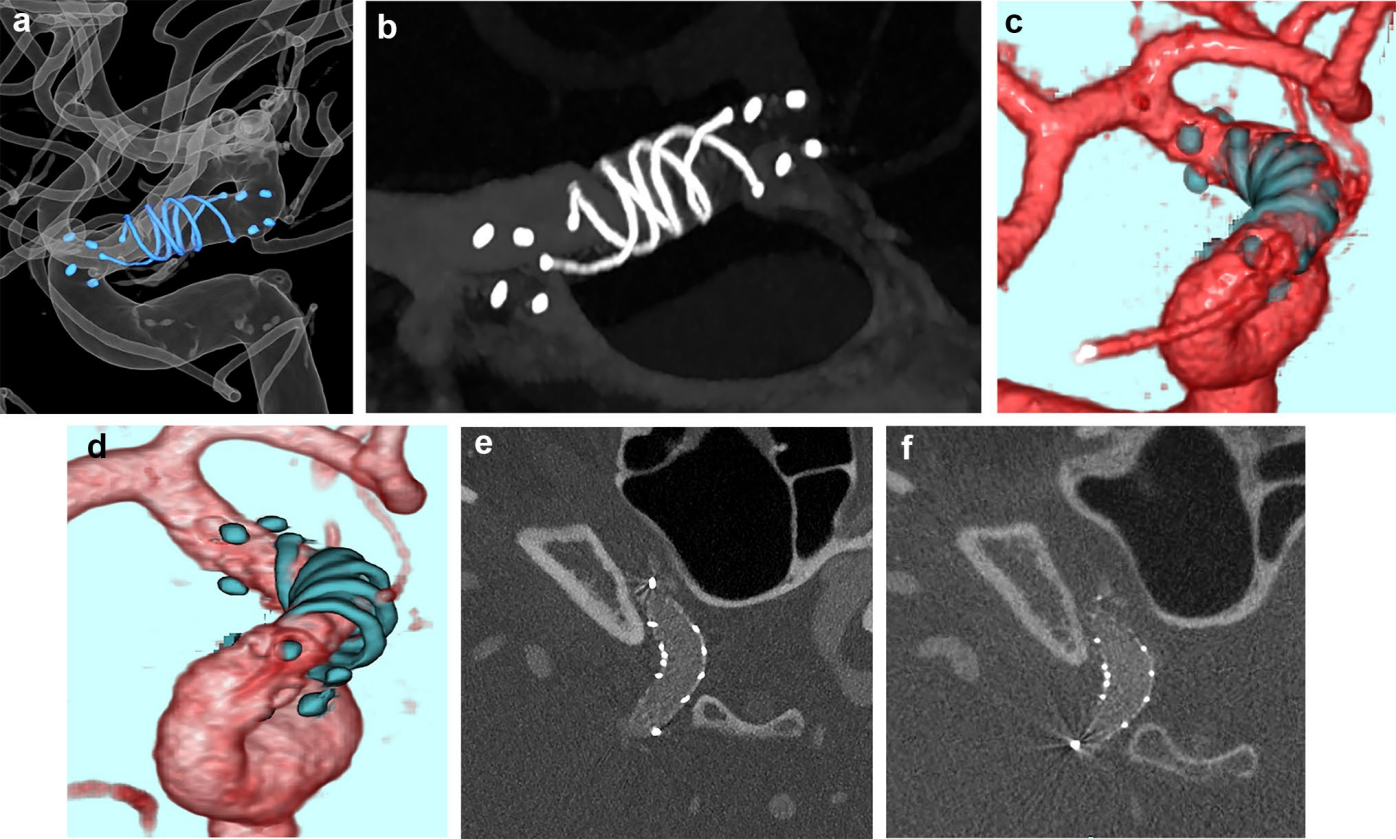
Cerebral aneurysm treated with a flow diverter stent. This case involved a woman in her 40 s who presented with a headache. The MRI revealed a 2.5-mm aneurysm with a broad neck (6 mm) on the anterior wall of the right internal carotid artery at the C2 segment. To treat the aneurysm, a flow diverter stent (FRED 4/18 mm) was implanted to treat the aneurysm (**a**). Both EID-CTA and PCD-CTA (kernel Hv89) were performed after flow diverter stenting. The PCD-CTA clearly showed the stent mesh, with image resolution comparable to that of traditional angiography (**b**). When comparing the 3D images from EID-CTA (**c**) and PCD-CTA (**d**), PCD-CTA was superior for evaluating the stent’s shape. The PCD-CTA also showed evidence of good apposition between the stent and vessel wall, with no thrombus formation in the stent (**e**: axial image). The resolution achieved with PCD-CTA was equal to that of angiography with cone-beam CT and diluted contrast media (**f**: axial image). *3D* three-dimensional, *CT* computed tomography, *MRI* magnetic resonance imaging, *EID-CTA* energy-integrating detector-computed tomography angiography, *PCD-CTA* photon-counting detector-computed tomography angiography

Furthermore, cone-beam CT (CBCT) imaging with angiography using diluted contrast media is a useful imaging tool for evaluating stent apposition and in-stent thrombosis after flow diverter stent implantation [21]. Herein, we present a case in which PCD-CTA was performed after flow diverter stent implantation, and the images by PCD-CTA were comparable to those obtained by CBCT angiography (Fig. 2). Many kernels are available for PCD-CT, and their functions suitable for the delineation of both the stent itself and the stent lumen have not yet been determined. A high kernel is useful for stent delineation because it allows a detailed evaluation of the mesh itself. Moreover, the image quality is nearly equivalent to that of CBCT; however, more cases must be accumulated to validate the evaluation of the intima within the stent.

### Spinal hemangioblastoma

Hemangioblastomas are highly vascular tumors with well-defined blood vessels that include feeding arteries and draining veins [22]. The location is determined as intramedullary, both intramedullary and extramedullary, intradural extramedullary, and epidural. In a study, two-thirds of the hemangioblastomas were solitary, whereas one-third was associated with von Hippel–Lindau disease [23]. The role of CTA in the diagnosis of hemangioblastoma involves the evaluation of the contrast effect of the tumor and the detection of the presence of feeding arteries and draining veins.

In angiographic findings of hemangioblastomas, the feeding arteries comprise the anterior and posterior spinal arteries, whereas the draining veins include the anterior and posterior spinal veins and the radiculomedullary vein [23]. Furthermore, it is important to identify the feeding vessels to embolize prior to tumor resection.

**Fig. 3:**
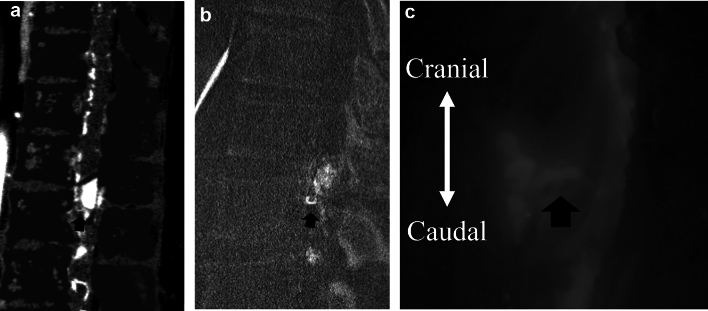
Spinal hemangioblastoma. A case of a male patient in his 60 s with lower back pain, gait disturbances, and pain in both the lower limbs. MRI T2WI showed an iso-intensity mass and flow voids around the mass. The PCD-CTA shows several masses (max 18 mm) with strong contrast effects with dilated blood vessels flowing into or out of the mass and a tiny curve vessel extending anteriorly from the mass in the sagittal view (**a**). The slab MIP image sagittal view of 3D angiography (**b**) shows a feeder from the anterior spinal artery and a draining vessel from the masses, which was almost equal to PCD-CTA. Intraoperative findings show a feeder from the anterior spinal artery, in contrast to the shiny structure in the ICG (**c**). *MRI* magnetic resonance imaging, *T2WI* T2-weighted imaging, *PCD-CTA* photon-counting detector-computed tomography angiography, *ICG* indocyanine green

Compared with magnetic resonance imaging (MRI), CTA enables a wider range of evaluation by facilitating the identification of feeding arteries originating far from the tumor. We presented a representative case of a hemangioblastoma with a feeding artery and dilated draining veins that were identified by PCD-CTA, and these were compared with the selective catheter spinal angiography and intraoperative findings (Fig. 3).

### Spinal hemangioma

Hemangiomas are not considered vascular neoplasms, but rather hamartomas or malformations of the microcirculation. Most spinal cord hemangiomas originate from the vertebral body and are typically asymptomatic [24]. Spinal cord hemangiomas without vertebral involvement are rare. In a review of primary spinal cord tumors, 57.2% were schwannomas, 11.6% were meningiomas, 8.0% were ependymomas, 4.0% were hemangiomas, 3.4% were hemangioblastomas, 3.4% were neurofibromas, and 1.3% were astrocytomas [25]. Intra- and extramedullary hemangiomas are rare [26]. Primary epidural cavernous hemangiomas have been misdiagnosed preoperatively as schwannomas, meningiomas, and arachnoid cysts, and the dural tail sign was positive in 30% of these hemangiomas [27]. Hemangiomas are classified according to the predominant vascular channel type (capillary, cavernous, arteriovenous, or venous) [28]. Although the role of angiography remains to be determined, it is useful for confirming the absence of dilated guiding veins, which are seen in hemangioblastomas, and for visualizing the vascular relationship between the lesion and the spinal vasculature to avoid significant intraoperative risks [29, 30].

**Fig. 4:**
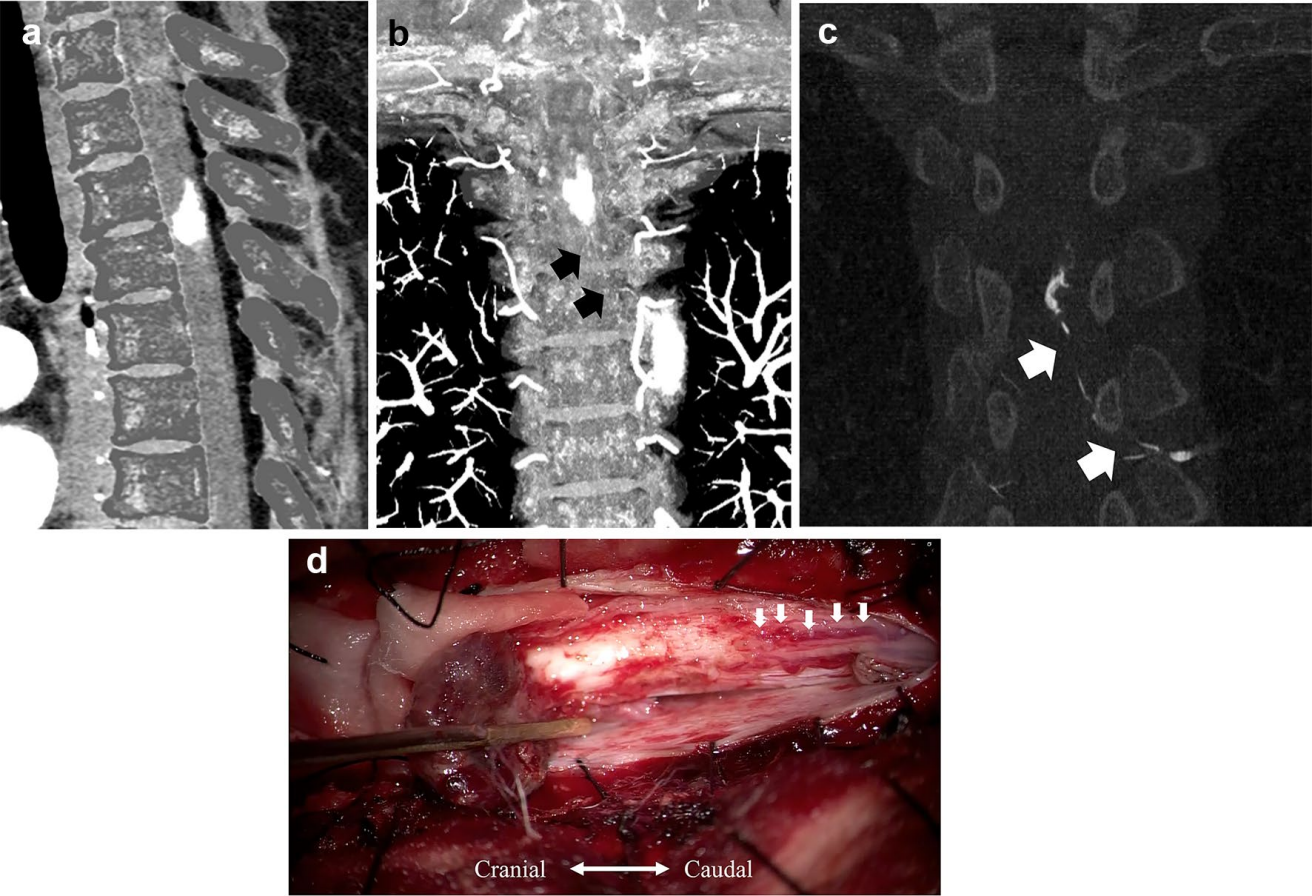
Spinal hemangioma. A case of a female patient in her 50 s with left scapular pain that extended to the lumbar region and cardiac fossa, gait disturbances, and rectal disturbances. Gd-T1WI showed a mass with a strong contrast effect in the spinal canal. On the sagittal images, a dural tail sign appeared, whereas axial images revealed an intramedullary tumor. A flow-void-like low-intensity signal was observed within the mass. PCD-CTA (kernel Qr60) images, including a sagittal view (**a**) and a coronal view (**b**), along with a coronal MIP image of the left Th5 from 3D angiography (**c**), show a 17-mm mass with a strong contrast effect located at Th3-4 of the spinal canal. A radiculopial artery branching from the intercostal artery at the left Th5 level flows into the posterior spinal artery and supplies the tumor. Intraoperative findings show several vessels entering the tumor (**d**), which is consistent with the PCD-CTA and angiography findings. The case was histopathologically diagnosed as a capillary hemangioma. *T1WI* T1-weighted imaging, *PCD-CTA* photon-counting detector-computed tomography angiography, *3D* three-dimensional, *Th* thoracic level

Here, we present a case in which we were able to preoperatively delineate a very small vessel inflow that was consistent with the angiography and intraoperative findings. Despite the very strong contrast effect, a small feeder could be delineated, and the absence of a dilated guiding vein helped rule out hemangioblastoma (Fig. 4).

### Spinal meningioma

Spinal meningiomas are the most common primary spinal cord tumors in adults and originate from leptomeningeal arachnoid cells [31]. Typically, spinal meningiomas are well-defined, intradural extramedullary tumors with homogeneous post-contrast enhancement on both CT and MRI. The thickening of the dura mater, with a peritumoral contrast effect, is a diagnostic criterion and is used to differentiate meningiomas from other lesions, such as schwannomas.

**Fig. 5:**
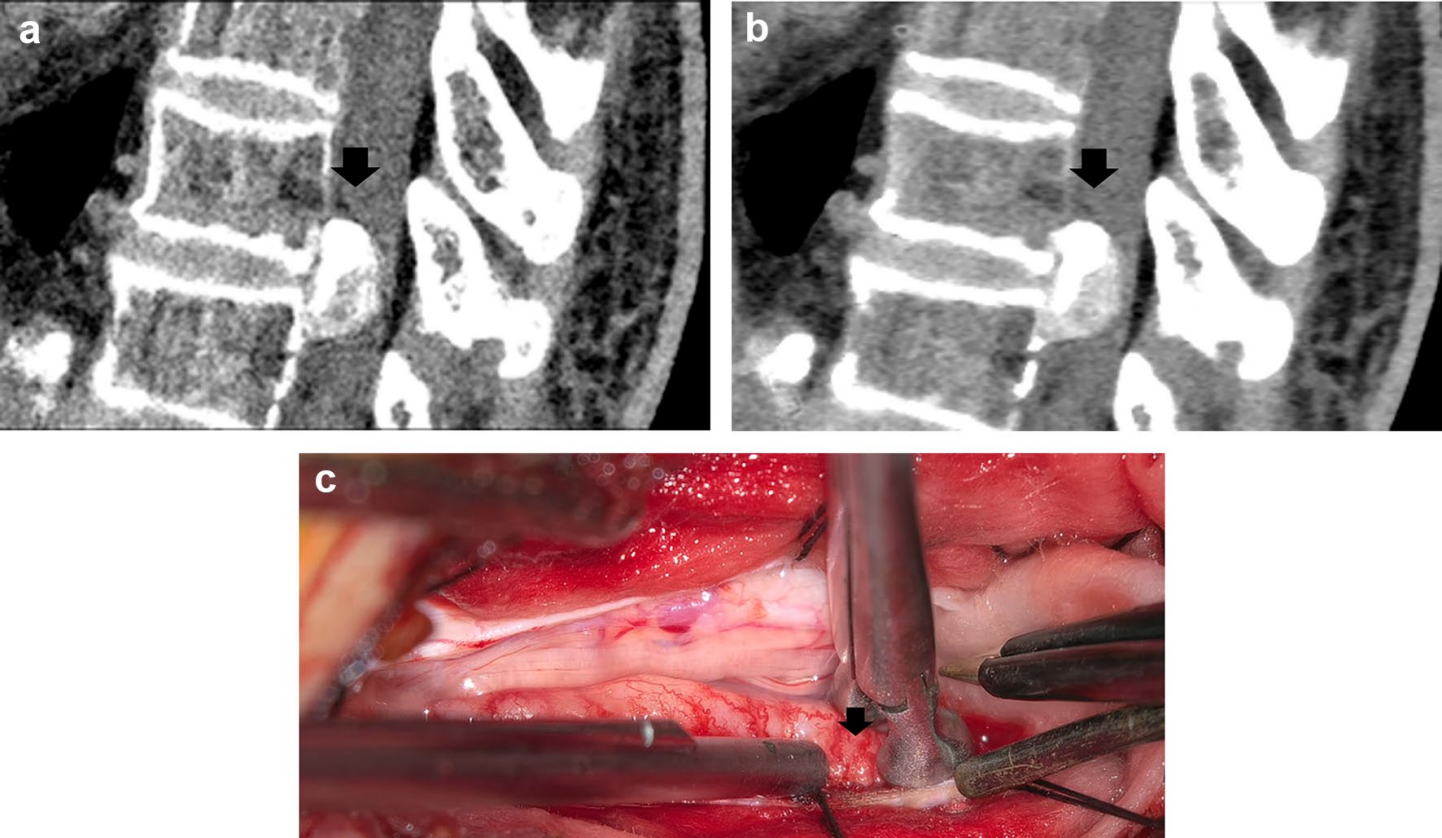
Spinal meningioma. In this case of a female patient in her 50 s with numbness in the plantar region, MRI T1WI showed an iso-intensity mass and T2WI showed a low-intensity mass at the Th10–11 level in the spinal canal. Gd-T1WI sagittal images showed strong homogeneous enhancement and a dural tail sign. PCD-CTA (kernel Qr60 and Qr44) revealed a 21-mm mass with homogeneous enhancement on the right side of the spinal canal, a dural tail sign, and coarse calcification. In contrast to Qr60 and Qr44, the coarse calcification of Qr60 (**a**) is more clearly delineated than that of Qr44 (**b**). Intraoperative findings show granular calcification mixed with the tumoral tissues (**c**). The tumor was elastic and hard. *MRI* magnetic resonance imaging, *T1WI* T1-weighted imaging, *T2WI* T2-weighted imaging, *PCD-CTA* photon-counting detector-computed tomography angiography

In addition to assessing tumor morphology and contiguous contrast effects, CT has a role in assessing calcification. Understanding the degree of calcification within the meningioma is important because calcification increases the difficulty of intraoperative tumor manipulation and the removal of dural adhesions [32]. In cardiovascular PCD-CTA, PURE calcium is used to remove iodine from the coronary CTA, and this leaves only coronary artery calcium because of the combination of high temporal and spatial resolutions and spectral data acquisition. In spinal PCD-CTA, this analysis can be used to identify intratumoral calcifications easily (Fig. 5).

### Glioblastoma

Glioblastoma is the most common primary brain tumor in adults and has the poorest prognosis. The fifth edition of the World Health Organization Classification of Tumors of the Central Nervous System (2021) defines glioblastoma as a diffuse astrocytic tumor of adults that must be IDH wild type. Malignant gliomas, particularly glioblastomas, show a characteristic capillary blush with early draining veins visible during the arterial phase of selective cerebral angiography, known as early venous drainage, indicating the presence of arteriovenous fistulae. In glioblastomas, a study, in which ^99m^Tc-labeled microparticles (macroaggregated albumin) were injected selectively transarterially into the tumor and brain and lung scintigraphy were performed, found that the majority of injected microparticles bypassed the tumor and reached the lungs: the average shunt index was 67% (range 47–89%). This finding confirms the importance of intra-tumor arteriovenous shunting [33].

**Fig. 6:**
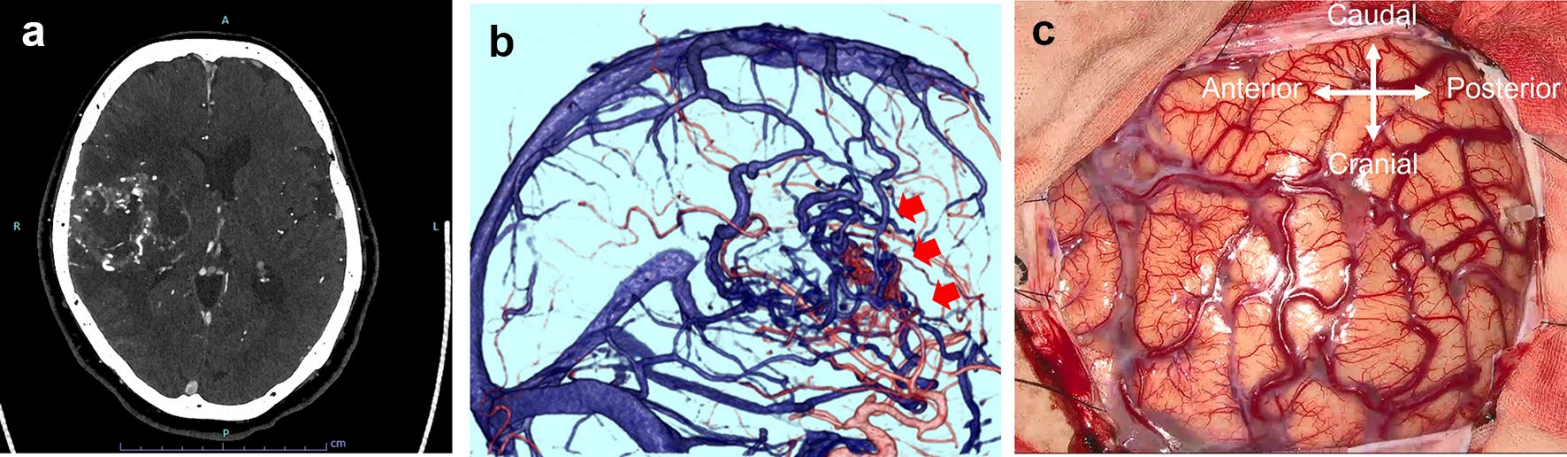
Glioblastoma. The case is of a woman in her 70 s. A few months prior, she fell and bruised her knee. Since then, she experienced difficulties in walking, and because of the progression of her symptoms, she visited a hospital. PCD-CTA (axial image: kernel Hv60) showed a ring-enhanced mass in the right fronto-parieto-temporal lobe (**a**). There were numerous microvessels in the wall of the mass, suggesting a hypervascular tumor with early venous filling and an AV shunt (**a**, **b**). Glioblastoma was suspected, and surgery was performed. Intraoperative view reveals a red cortical vein (**c**). *PCD-CTA* photon-counting detector-computed tomography angiography, *AV* arteriovenous

The finding of highly vascularized tumors, especially the existence of an arteriovenous shunt on CTA, is important for the diagnosis of glioblastoma and also predicts the risk of bleeding during surgery (Fig. 6).

## Summary

In this review, we updated the information from conventional reviews and presented SEAVF, a giant cerebral aneurysm treated with flow diverter, spinal hemangioblastoma, spinal hemangioma, spinal meningioma, and glioblastoma, and evaluated how well the cranial and spinal PCD-CTA approaches the accuracy of angiographic and intraoperative findings.

## Conclusion

The clinical PCD-CT provides high spatial resolution imaging with reduction of artifacts. The current role of CTA is primarily complementary to angiography. Substituting high-resolution images with PCD-CT for angiography can be beneficial to patients in future.
